# Clinical adjacent segment pathology after anterior cervical discectomy, with and without fusion, for cervical degenerative disc disease: A single center retrospective cohort study with long-term follow-up

**DOI:** 10.1016/j.bas.2022.100869

**Published:** 2022-01-22

**Authors:** Valérie N.E. Schuermans, Anouk Y.J.M. Smeets, Nienke P.M.H. Wijsen, Inez Curfs, Toon F.M. Boselie, Henk van Santbrink

**Affiliations:** a Department of Neurosurgery, Maastricht University Medical Center+, P. Debyelaan 25, 6229 HX, Maastricht, the Netherlands; b Department of Neurosurgery, Zuyderland Medical Center, Henri Dunantstraat 5, 6419 PC, Heerlen, the Netherlands; c Faculty of Health, Medicine and Life Sciences, Maastricht University, Universiteitssingel 40, 6229 ER, Maastricht, the Netherlands; d Department of Orthopaedic Surgery and Traumatology, Zuyderland Medical Center, Henri Dunantstraat 5, 6419 PC, Heerlen, the Netherlands; e CAPHRI School for Public Health and Primary Care, Maastricht University, Universiteitssingel 40, 6229 ER, Maastricht, the Netherlands

**Keywords:** Cervical degenerative disc disease, Adjacent segment pathology, Anterior cervical discectomy and fusion, Cervical radiculopathy, Cervical myelopathy

## Abstract

**Introduction:**

Clinical adjacent segment pathology (CASP) continues to be a cause of concern after anterior surgical treatment for single- or multilevel cervical degenerative disc disease (CDDD). The current literature reports inconsistent incidence rates and contended risk factors in the development of CASP.

**Research question:**

The aim is to determine the incidence of additional CASP-related surgeries after anterior cervical discectomy with fusion (ACDF) or without fusion (ACD) for CDDD. Secondary outcomes include risk factors for the development of CASP and long-term clinical outcomes.

**Materials & methods:**

This is a single-center, retrospective cohort study with a long-term follow up. Patients undergoing ACD(F) for CDDD between January 2012 and December 2019 were included.

**Results:**

A total of 601 patients were included, with an average follow-up period of 5.0 years. Most patients underwent ACDF with stand-alone cages (87.7%). CASP developed in 58 (9.7%) patients, 41 (70.7%) of which required additional adjacent level surgery. ACD significantly accelerated the development of CASP. The C2–C7 Cobb angle appeared less lordotic upon early post-operative imaging in ACDF patients that later-on developed CASP. Baseline degeneration at the index level and adjacent levels was not significantly different between patients with and without CASP.

**Discussion & conclusion:**

In this retrospective cohort, we observe a relatively low rate of additional surgery for CASP in ACDF with stand-alone cages. We suggest that surgical technique, fusion, segmental kyphosis and natural degeneration play a multifactorial role in the development of CASP. Complication rates were low and clinical outcomes were similar for all techniques used.

## Highlights

• CASP developed in 9.7% patients, of whom 70.7% required additional adjacent level surgery.
• Very low incidence of symptomatic cage subsidence after ACDF with stand-alone cages.
• Discectomy alone significantly accelerated the development of CASP.
• Baseline degeneration was not associated with the development of CASP.

## Abbreviations

ACD: Anterior Cervical Discectomy
ACDF: Anterior Cervical Discectomy with Fusion
ACDA: Anterior Cervical Discectomy and Arthroplasty
BMI: Body Mass Index
CASP: Clinical Adjacent Segment Pathology
CDDD: Cervical Degenerative Disc Disease
KS: Kellgren Score
RASP: Radiological Adjacent Segment Pathology
STROBE: STrengthening the Reporting of OBservational studies in Epidemiology
WMO: Medical Research Involving Human Subjects Act

## 1 Introduction

One of the most common surgical treatment options for radiculopathy and/or myelopathy due to single- or multilevel cervical degenerative disc disease (CDDD) is anterior cervical discectomy, either with fusion (ACDF) or without (ACD) (Korinth, 2008). While both techniques show good, and similar, clinical outcomes in the short term (Fehlings et al., 2015; Xie and Hurlbert, 2007; Joo et al., 2010), patient-reported satisfaction gradually drops over the long term (Donk et al., 2017; Nandoe Tewarie et al., 2007). This is thought to result from the development of new complaints of radiculopathy and/or myelopathy at levels adjacent to the index level, also known as adjacent segment pathology (Riew et al., 2012). This occurs at an estimated cumulative rate of 1.6%–4.2% per year (Riew et al., 2012). The prevalence of patients requiring additional adjacent segment surgery is estimated to be 0.8% per year, although there is inconsistency among the reported rates (Gore and Sepic, 1984; Williams et al., 1968; Bohlman et al., 1298; Hilibrand and Robbins, 2004; Hilibrand et al., 1999; Gore and Sepic, 1998; Lawrence et al., 2012), likely a result of heterogeneous definitions and differences in diagnostic criteria for adjacent segment pathology. For example, some reports include radiologic changes without clinical symptoms, while others include re-operations at the index level as additional surgery. A distinct definition that distinguishes *radiologic* adjacent segment pathology (RASP) from *clinical* adjacent segment pathology (CASP) is recommended (Riew et al., 2012).

The underlying mechanism of CASP is thought to be compensation for the loss of motion in the fused segment, resulting in overstraining of the adjacent segments in addition to progression of natural degeneration (Helgeson et al., 2013; Seo and Choi, 2008; Eck et al., 2002). Several studies have shown higher degrees of RASP in levels adjacent to previously fused segments in comparison to segments treated with motion-preserving techniques (Garrido et al., 2011; Robertson et al., 2005; Kim et al., 2009). Indeed, this theory is supported by findings in patients with congenital cervical spinal fusions that show higher degrees of both RASP and CASP (Song et al., 2011; Guille et al., 1995). However, controversy remains. Some studies argue against the role of fusion surgery in the development of CASP, and instead suggest that natural degeneration is the key factor (Hilibrand et al., 1999; Helgeson et al., 2013). They postulate that patients that undergo surgery for CDDD have already proven that their discs are susceptible to degeneration (Helgeson et al., 2013). Even though all treatment techniques show equal short-term clinical outcomes, differences in the incidence of CASP can influence the long-term clinical outcomes. Hence, the extent to which radiological parameters, surgery-induced fusion and natural degeneration play a role in the development of RASP and CASP remains unknown.

The socioeconomic impact of CDDD is high, since it predominantly affects the working population (Radhakrishnan et al., 1994; Salemi et al., 1996; Boogaarts and Bartels, 2015). Secondary absenteeism, hospitalization and additional surgeries as a consequence of CASP further increase the socioeconomic impact. In order to limit the occurrence of CASP, it is important to determine the factors contributing to its development. Therefore, the aim of this study is to determine the incidence and risk factors of additional CASP-related surgeries after anterior cervical surgery in patients with CDDD presenting with radiculopathy and/or myelopathy. This is a single-center, retrospective cohort study with long-term follow-up.

## 2 Materials & methods

### 2.1 Design

This retrospective cohort study was conducted in a regional spinal surgery center – the Zuyderland Medical Center in the Netherlands. A consecutive series of patients that underwent anterior cervical decompression surgery between January 2012 and December 2019 were included by chart review. Patients were contacted in July–September 2020 to retrieve long-term (at least one year) follow-up data. This study has been approved by the Medical Ethical Committee (METCZ20200004) and was conducted according to the principles enshrined in the Declaration of Helsinki and in accordance with the Medical Research Involving Human Subjects Act (WMO). This manuscript is written in accordance with the STrengthening the Reporting of OBservational studies in Epidemiology (STROBE) guidelines (Cuschieri, 2019).

### 2.2 Population

Adult patients who underwent surgery for symptomatic single- or multilevel radiculopathy and/or myelopathy due to CDDD were eligible for inclusion. Patients were included if either of the following surgical techniques was used: ACD (i.e., without interbody spacer), or ACDF (interbody spacer with or without plate-construct). Treatment should be well-documented, and adequate follow-up data – defined as at least one outpatient follow-up visit – should be available in electronic patient records. Only patients with degenerative causes of radiculopathy and/or myelopathy in the cervical spine were included.

### 2.3 Data collection

A search of medical treatment codes was conducted to identify all patients undergoing anterior cervical decompression surgery. Data were collected through chart review and stored in a coded database. Baseline characteristics were collected, which included: gender, age, Body Mass Index (BMI), smoking, use of pain medication, indication and level of the initial surgery, degree of pre-operative degeneration according to the Kellgren-Lawrence Score (KS) (KELLGREN and LAWRENCE, 1958; Kettler and Wilke, 2006) [Table 1], surgical technique, surgical complications, cervical sagittal balance, fusion status and clinical outcomes according to the Odom Criteria (ODOM et al., 1958). KSs were assessed retrospectively by two independent reviewers *(NW, VS)* for each independent cervical level on pre-operative radiographs (X-rays). In case of conflict, a third reviewer (*AS*) was consulted to reconcile differences in scores. For the assessment of fusion, we only included imaging that was made more than 1 year after the index surgery. The cervical sagittal balance is assessed by measuring the Cobb angle of C2–C7 and the Cobb angle of the index segment. Sagittal alignment was only assessed on lateral, standing X-rays. A kyphotic Cobb angle is scored as being a negative value, whereas a lordotic Cobb angle is scored as a positive value. In our practice, standard post-operative imaging was performed 1 day after surgery. Later follow-up imaging was only performed upon indication; only post-operative imaging made before the additional surgery for CASP is assessed.

**Table 1:** Kellgren score.

Grade	Description
0	No signs of degenerative disc disease.
1	Minimal anterior osteophytes.
2	Definite anterior osteophytosis with possible narrowing of the disc space and some sclerosis of vertebral plates.
3	Moderate narrowing of the disc space with definite sclerosis of vertebral plates and osteophytes.
4	Severe narrowing of the disc space with definite sclerosis of vertebral plates and multiple large osteophytes.

This is a score ranging from 0 to 4 to indicate the level of degeneration at every level of the cervical spine.

The Odom Criteria ranged from “Excellent” (indicating complete amelioration of complaints without any limitations in daily life), to “Poor” (indicating unchanged or exacerbated complaints with persistent limitations in daily life). Notably, this study aims to determine the rate of clinical adjacent segment disease only; it does not aim to determine radiological success. As post-operative imaging is not standard in the study center, post-operative alignment and fusion rates were not assessed as secondary outcomes.

### 2.4 Outcome measures

The primary outcome is the occurrence of additional adjacent segment surgery for CASP. CASP was defined as the presence of newly developed symptomatic cervical radiculopathy and/or myelopathy on a level adjacent to the initial surgery, confirmed by corresponding findings upon magnetic resonance imaging (MRI). It should be noted that *radiological* adjacent segment pathology (RASP) is differentiated from *clinical* adjacent segment pathology (CASP) by the presence of clinical symptoms that can be attributed to the degenerative changes for the latter. Patients were only considered as having CASP when the index surgery initially provided improvement of symptoms. Additional adjacent segment surgery for CASP was defined as surgery for radiculopathy and/or myelopathy at a segment adjacent to the level of initial surgery. Notably, neck pain itself is not considered as a surgical indication in our national guidelines ((NVvN) Nederlandse Vereniging voor Neurochirurgie, 2010). Re-operations at the index level and at levels not adjacent to the initially operated level were not considered as additional surgery for CASP. Therefore, re-operations at the index level are discussed separately. The total follow-up time was calculated from the date of the index surgery to the time of the last follow-up.

Patients were contacted by telephone between July and September 2020. Patients were asked two standardized questions: the validated Odom Criteria, and whether they had undergone additional surgery in another hospital since the last outpatient contact in the study center. Symptoms were not assessed during the phone call. When patients could not be reached, the last documented neurosurgical or neurological outpatient visit was considered as the last follow-up contact.

Secondary outcome measures were: potential risk factors for the development of CASP (gender, smoking, BMI, age, level(s) of surgery and baseline level of degeneration), and clinical outcome after surgery using the Odom's Criteria at the long-term follow-up contact. It should be noted that for radiculopathy, a clinical outcome was only considered good if the pre-operative complaints improved. For myelopathy, a clinical outcome was also considered good if complaints were stabilized.

### 2.5 Data analysis

Statistical analyses were carried out using IBM SPSS statistics 27 (Cooperation). Descriptive data were generated for all variables. Univariate analysis was performed for baseline characteristics. The independent samples *t*-test, Chi-Square and Fisher's exact test were used to determine statistical differences between groups. Survival and Hazard ratios are presented in a Kaplan-Meier curve; estimates were determined using a Log-Rank test. Multivariate Cox proportional hazard models were used to determine whether time to additional adjacent segment surgery was associated with pre- and perioperative risk factors. A p-value below 0.05 was considered statistically significant.

## 3 Results

### 3.1 Baseline characteristics

**Fig. 1:**
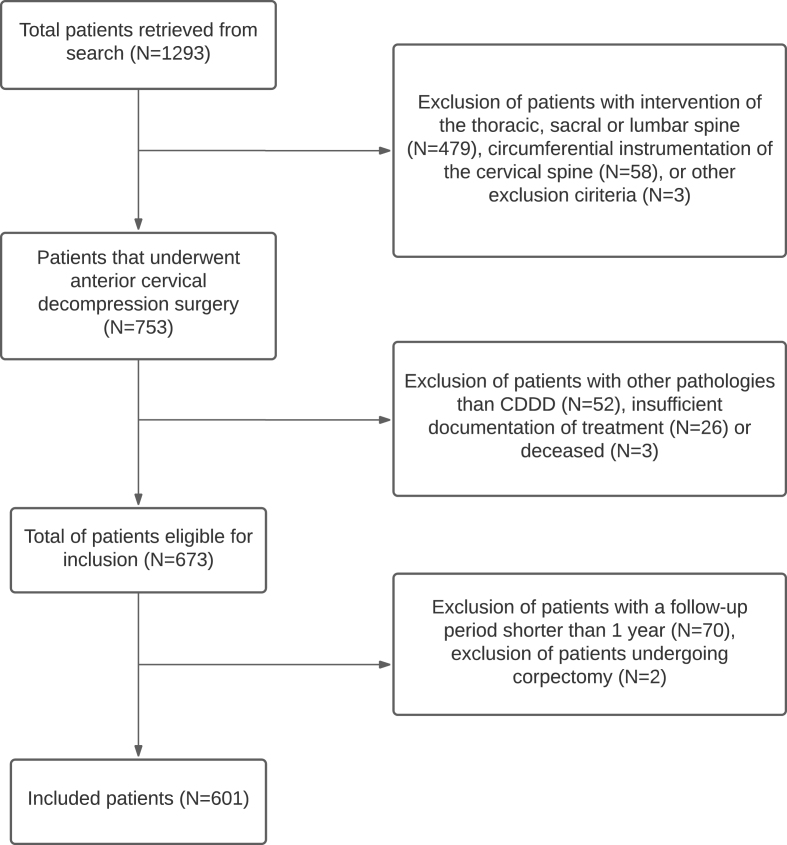
Flowchart of patient selection. ACD ​= ​Anterior Cervical Discectomy, ACDF ​= ​Anterior Cervical Discectomy and Fusion, CDDD = Cervical Degenerative Disc Disease

Charts of 1293 patients with 1401 interventions were reviewed. From these, 673 patients were eligible for inclusion [Fig. 1]. Two patients undergoing corpectomy were excluded to obtain a more homogenous population. Patients were excluded if they had a follow-up time less than 1 year (N ​= ​70). In total, 601 patients were included, of whom 471 (78.4%) were successfully contacted to retrieve long-term follow-up data. Of the remaining 130 (21.6%) patients, data from the last outpatient visit were retrieved from the charts. This resulted in a mean total follow-up time of 5.0 years, ranging from 1.0 to 32.7 years.

When assessing patient eligibility, 23 patients had overlapping treatment codes and appeared to have an ACD(F) before 2012. This explains the follow-up time exceeding 8 years. We included those patients in our cohort as we estimated a low risk of selection bias. Moreover, they did not have a substantial influence on the mean follow-up time.

Baseline characteristics are presented in Table 2. The indication for index surgery was isolated radiculopathy in 57.2% of the patients (N ​= ​344), isolated myelopathy in 34.4% of the patients (N ​= ​207), and mixed symptoms of myeloradiculopathy in 8.3% of the patients (N ​= ​50). ACDF without plating was performed in most patients (87.7%). The most frequently operated segment was C5C6, followed by C6C7 [Table 2].

**Table 2:** Baseline characteristics.

Baseline characteristics	No CASP (N ​= ​560)	CASP (N ​= ​41)	Sig. (2-sided) [95%CI]
Gender			
Female	247 (44.1%)	21 (51.2%)	0.418
Male	313 (55.9%)	20 (48.8%)	
Age	53 ​± ​10.9	49 ​± ​9.1	0.025 [-7.363; −0.496]
BMI	27.1 ​± ​4.7	27.1 ​± ​4.9	0.951 [-1.455; 1.549]
Smoking	254 (45.4%)	27 (65.9%)	**0.011∗**
Indication of initial surgery			
Radiculopathy	323 (57.7%)	21 (51.2%)	0.577
Myelopathy	192 (34.3%)	15 (36.6%)	
Both	45 (8.0%)	5 (12.2%)	
Pre-operative duration of complaints			
<6 weeks	12 (2.1%)	1 (2.4%)	0.950
6 weeks – 3months	41 (7.3%)	2 (4.9%)	
3 months - 1 year	300 (53.6%)	22 (53.7%)	
>1 year	206 (36.8%)	14 (34.1%)	
Unknown	1 (0.2%)	2 (4.9%)	
Technique of initial surgery			
ACD	9 (1.6%)	14 (34.1%)	**<0.001∗**
ACDF	502 (89.7%)	25 (61.0%)	
ACDF ​+ ​plating	46 (8.2%)	2 (4.9%)	
Hybrid surgery	3 (0.5%)	0 (0%)	
Level of initial surgery	*Including multilevel surgeries (N* ​= ​*695 operated levels)*	*Including multilevel surgeries (N* ​= ​*46 operated levels)*	
C3C4	60 (8.6%)	3 (6.5%)	0.608
C4C5	95 (13.7%)	6 (13.0%)	0.831
C5C6	318 (45.8%)	21 (45.7%)	0.517
C6C7	211 (30.4%)	13 (28.3%)	0.506
C7T1	11 (1.6%)	3 (6.5%)	0.063
Levels of initial surgery			
Single	432 (77.1%)	36 (87.8%)	0.223
2-level	122 (21.8%)	5 (12.2%)	
>2 levels	6 (1.1%)	0 (0%)	
Perioperative complications	10 (1.8%)	0 (0%)	0.635

Significant difference between groups with and without CASP is determined using univariate analysis, indicated with bold values and an asterisk (∗). CASP = Clinical Adjacent Segment Pathology, ACD ​= ​Anterior Cervical Discectomy, ACDF ​= ​Anterior Cervical Discectomy and Fusion**.**

### 3.2 Primary outcome: additional surgery for CASP

**Fig. 2:**
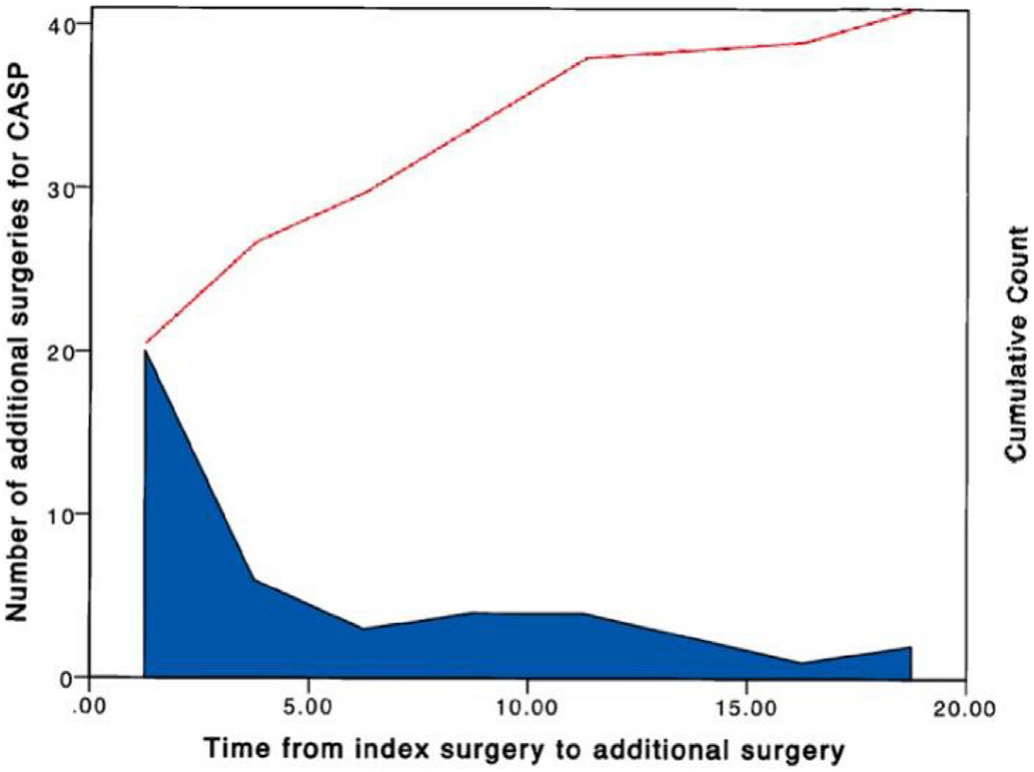
**Time from initial surgery to additional** adjacent segment **surgery for CASP**. This histogram represents the number of patients undergoing additional adjacent segment surgeries for CASP divided per time in years. Frequency represents the number of patients.

Of the 601 included patients, 58 (9.7%) developed new symptoms of radiculopathy and/or myelopathy due to CASP at the time of last follow-up. Of these 58 patients, 41 required additional adjacent segment surgery (6.8%), and 21 (51.2%) of these underwent additional adjacent segment surgery within 2.5 years [Fig. 2]. The remaining 17 patients (2.8%) were symptomatic without requiring additional adjacent segment surgery [Table 3]. This represents an incidence rate of CASP of 3.2 per 10,000 person years, and an incidence rate of additional adjacent segment surgery as a consequence of CASP of 2.3 per 10,000 person years.

**Table 3:** Primary outcome measurements.

**Primary outcome: CASP (N** ​= ​**601) (%)**	
CASP	58 (9.7%)
Additional surgery for CASP	41 (6.8%)
**Surgical Technique used for additional surgery for CASP (N** ​= ​**41)**	
ACD	2 (4.9%)
ACDF	24 (58.5%)
ACDF ​+ ​plating	6 (14.6%)
Corpectomy ​+ ​plating	1 (2.4%)
Circumferential spondylodesis	3 (7.3%)
Dorsal foraminotomy	5 (12.2%)
**Level of additional surgery in relation to primary surgery (N** ​= ​**41)**	
Above	16 (39.0%)
Below	19 (46.3%)
Both above and below	6 (14.6%)

CASP = Clinical Adjacent Segment Pathology, ACD ​= ​Anterior Cervical Discectomy, ACDF ​= ​Anterior Cervical Discectomy and Fusion**.**

The surgical techniques for the secondary surgery are presented in Table 3. Additional adjacent segment surgery was performed above the level of the index surgery in 16 patients, below in 19 patients, and both above and below in 6 patients [Table 3].

### 3.3 Secondary outcomes

#### 3.3.1 Risk factors

##### 3.3.1.1 Baseline characteristics

**Fig. 3:**
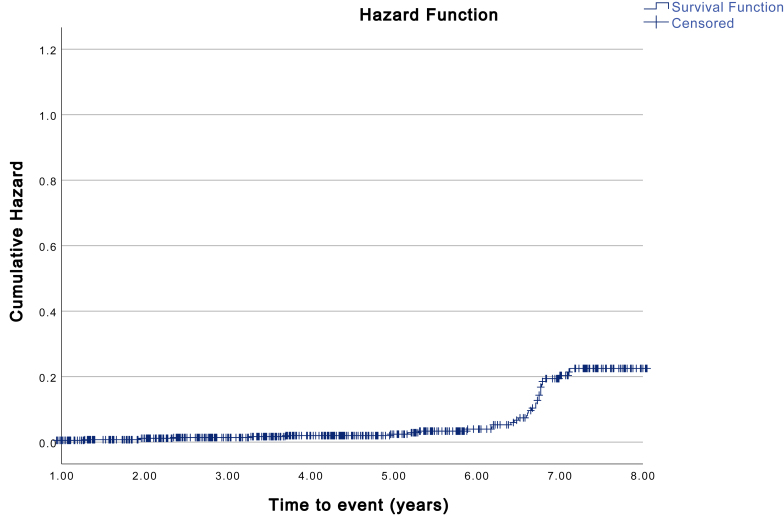

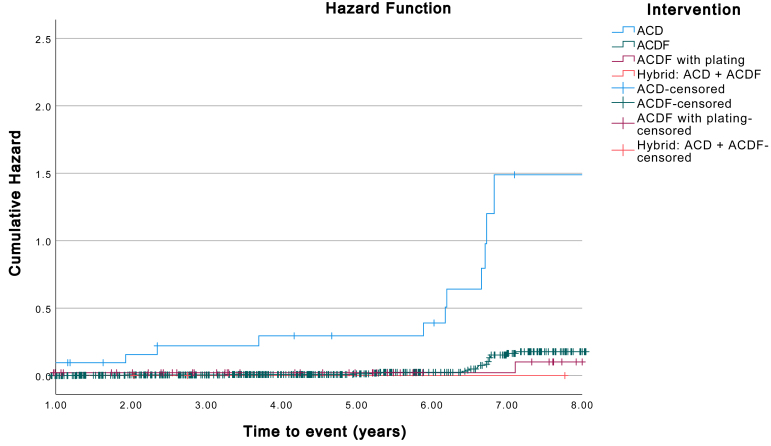
a. Kaplan-Meier Hazard Function X-axis depicting time to additional adjacent segment surgery for CASP, Y-axis depicting Cumulative Hazard Ratio. b. Kaplan-Meier Hazard Function. X-axis depicting time to additional adjacent segment surgery for CASP with type of intervention as a factor. Y-axis depicting Cumulative Hazard Ratio. Survival and Hazard functions are cut at 8 years follow-up time to prevent skewing by shorter follow-up times.

Fig. 3 depicts the Kaplan-Meier Hazard curve for additional adjacent segment surgery for CASP. Estimates show a significantly lower probability of CASP for patients that underwent ACDF compared to ACD as the initial surgery (p ​< ​0.001). The time-points marked as “censored” represent the end of the follow-up period of an individual. When comparing baseline characteristics between the groups with and without CASP, there was a significantly higher percentage of smokers in the CASP group (p ​= ​0.011). This difference was not significant in a multivariate analysis when correcting for confounding factors. No other covariates could be identified as a significant risk factor for additional adjacent segment surgery for CASP in the multivariate analysis. The incidence of CASP was similar between patients with single- and multilevel surgery.

#### 3.3.2 Baseline degeneration

The preoperative KS did not differ significantly at the index level, nor at one level above or below the index level for patients with and without CASP [Table 4].

**Table 4:** Kellgren Score at index and adjacent levels.

Pre-operative Kellgren's score	Mean of total group (N)	SD	No CASP	SD	CASP	SD	Significance p-value
Average of all levels	0.83 (N ​= ​591)	±0.72	0.82 (N ​= ​556)	±0.72	0.87 (N ​= ​35)	±0.73	0.695
Average of index level	1.85 (N ​= ​338)	±1.04	1.86 (N ​= ​319)	±1.04	1.63 (N ​= ​19)	±1.01	0.358
Average of the level **above** index level	1.00 (N ​= ​586)	±1.08	1.00 (N ​= ​552)	±1.07	1.16 (N ​= ​34)	±1.22	0.386
Average of the level **below** index level	1.11 (N ​= ​416)	±1.23	1.10 (N ​= ​394)	±1.22	1.27 (N ​= ​22)	±1.35	0.533

Number of patients is represented per parameter, as not all KSs were available. CASP = Clinical Adjacent Segment Pathology, SD = Standard Deviation.

##### 3.3.2.1 Fusion status

Post-operative imaging was available in 127 cases, of which 65 were X-rays and 22 were CT-scans. Of the remaining 40 patients, only post-operative MRI imaging was available. On average, fusion was assessed 4 years after index surgery (±3.6 years). The results of fusion assessment are presented in Table 5. Fusion did not differ substantially between those with CASP (80.6%) and without CASP (70.0%). There was no evident difference in fusion status between surgical techniques used. In this cohort, the presence of fusion was not correlated with the development of CASP, as assessed with cox-regression, corrected for the intervention and the imaging on which fusion was assessed.

**Table 5:** Fusion status.

Fusion assessment			Presence of fusion						
	No imaging (N ​= ​)	Available imaging (N ​= ​)	Yes (N ​= ​)	No (N ​= ​)	Inconclusive (N ​= ​)	Intervention (N ​= ​)	Yes (N ​= ​)	No (N ​= ​)	Inconclusive (N ​= ​)
**NO CASP**	464	96	72 (75.0%)	1 (1.0%)	23 (24.0%)	ACD (n ​= ​1)	1 (100%)	0	0
						ACDF (n ​= ​87)	64 (73.6%)	1 (1.1%)	22 (25.3%)
						ACDF ​+ ​plate (n ​= ​8)	7 (87.5%)	0	1 (12.5%)
**CASP**	10	31	25 (80.6%)	2 (6.5%)	4 (12.9%)	ACD (n ​= ​11)	9 (81.8%)	2 (18.2%)	0
						ACDF (n ​= ​19)	16 (84.2%)	0	3 (15.8%)
						ACDF ​+ ​plate (n ​= ​1)	0	0	1 (100%)

This table displays the fusion status of the available post-operative images. Fusion was only assessed upon post-operative imaging that was made more than 1 year after the index surgery. Fusion status is scored as “yes”, “no” or “inconclusive”, the latter pertaining to situations when the imaging quality was too low or fusion could not be assessed on magnetic resonance imaging (MRI). CASP = Clinical Adjacent Segment Pathology.

##### 3.3.2.2 Sagittal balance

Pre- and post-operative sagittal balance was assessed in all available imaging (Table 6). C2–C7 and segmental lordosis was similar between patients with and without CASP pre-operatively.

**Table 6:** Sagittal balance.

Sagittal balance	Pre-operative X-ray					Direct post-operative X-ray					Follow-up X-ray					
	Mean 1.97 years (±2.90 years)					Mean 1.35 days (±4.38 days)					Mean 1.5 ​y (±2.37 years)					
C2 – C7 lordosis																
	Mean	SD	Intervention	Mean	SD	Mean	SD	Intervention	Mean	SD	Mean	SD	Intervention	Mean	SD	
**No CASP**	9.14 (n ​= ​187)	±12.00	ACD	–	±12.05 ±11.89	8.55 (n ​= ​506)	±12.13	ACD	11.25 (n ​= ​4)	±11.67	8.50 (n ​= ​197)	±11.91	ACD	–	–	
			ACDF	9.09 (n ​= ​167)				ACDF	**8.16∗ (n** ​= ​**455)**	± ​**12.29**			ACDF	7.60 (n ​= ​164)	±12.11	
			ACDF ​+ ​plate	9.60 (n ​= ​20)				ACDF ​+ ​plate	12.20 (n ​= ​44)	±10.21			ACDF ​+ ​plate	12.97 (n ​= ​33)	±9.82	
**CASP**	17.38 (n ​= ​8)	±15.15	ACD	33.00 (n ​= ​1)	–	6.40 (n ​= ​25)	±9.09	ACD	28.00 (n ​= ​1)	–	9.52 (n ​= ​21)	±12.03	ACD	12.75 (n ​= ​4)	±9.96	
			ACDF	13.50 (n ​= ​6)	±15.57			ACDF	**4.46∗ (n** ​= ​**22)**	± ​**7.72**			ACDF	8.33 (n ​= ​15)	±10.74	
			ACDF ​+ ​plate	25.00 (n ​= ​1)	–			ACDF ​+ ​plate	15.00 (n ​= ​2)	±7.08			ACDF ​+ ​plate	12.00 (n ​= ​2)	±2.83	
**Segmental lordosis of index level**																
		**SD**	**Intervention**	**Mean**	**SD**	**Mean**	**SD**	**Intervention**	**Mean**	**SD**	**Mean**	**SD**	**Intervention**	**Mean**	**SD**	
**No CASP**	1.22 (n ​= ​186)	±5.54	ACD	–	±5.52 ±5.86	3.53 (n ​= ​522)	±5.49	ACD	4.00 (n ​= ​4)	±10.42	2.06 (n ​= ​199)	±6.12	ACD	–	–	
			ACDF	1.21 (n ​= ​166)				ACDF	**3.57∗ (n** ​= ​**470)**	± ​**5.48**			ACDF	1.78 (n ​= ​165)	±6.37	
			ACDF ​+ ​plate	1.30 (n ​= ​20)				ACDF ​+ ​plate	2.93 (n ​= ​45)	±5.34			ACDF ​+ ​plate	3.41 (n ​= ​34)	±4.59	
**CASP**	1.25 (n ​= ​8)	±5.25	ACD	−6.00 (n ​= ​1)	–	3.40 (n ​= ​25)	±4.97	ACD	−7.50 (n ​= ​2)	±3.54	0.90 (n ​= ​20)	±7.29	ACD	−5.00 (n ​= ​4)	±12.91	
			ACDF	1.83 (n ​= ​6)	±5.00			ACDF	**4.43∗ (n** ​= ​**21)**	±**3.97**			ACDF	2.29 (n ​= ​14)	±5.07	
			ACDF ​+ ​plate	5.00 (n ​= ​1)	–			ACDF ​+ ​plate	3.50 (n ​= ​2)	±2.12			ACDF ​+ ​plate	3.00 (n ​= ​2)	±0.00	

This table displays the measured Cobb's angles on pre-operative, direct post-operative and later post-operative X-ray images. Significant differences between groups (α ​< ​0.05) are marked in bold with an asterisk (∗).

Note: Numbers between rows do not always match because lower cervical vertebrae were not always visible upon available imaging. Consequently C2–C7 could not always be measured when the index level could be measured, or the other way around, when the index level was C7-T1, the C2–C7 slope could be measured, but not the index segment.

CASP = Clinical Adjacent Segment Pathology, SD= Standard Deviation.

Specifically, ACDF patients that developed CASP appeared to have significantly less C2–C7 lordosis after the index surgery, compared to those that did not develop CASP.

The influence of the Cobb angle on the development of CASP was assessed with Cox-regression analysis. Analyses were performed for each measurement separately to allow larger sample sizes, since listwise exclusion of Cobb angle excludes too many patients. All models were corrected for the intervention used. Means for subgroups were compared with both paired and independent t-tests.

#### 3.3.3 Odom Criteria

**Fig. 4:**
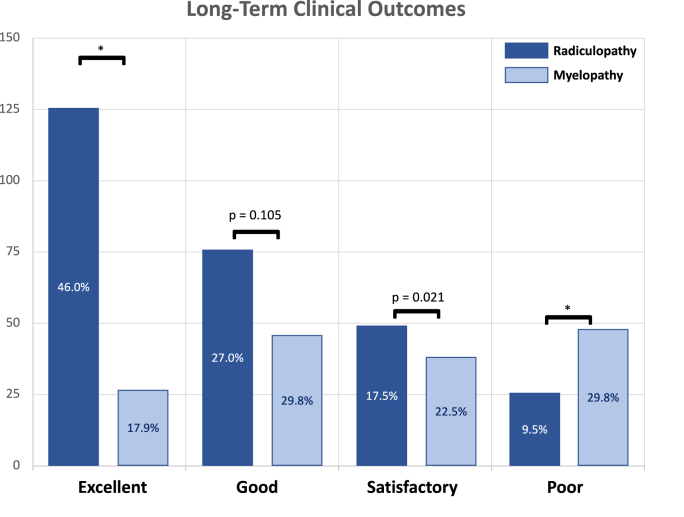
Long-term clinical outcomes. This table represents the Odom's Criteria of patients that were reached for long-term follow-up. Statistical significance is illustrated with an asterisk “∗”.

For long-term clinical outcomes, the Odom Criteria were available for 471 out of 601 patients (78.4%) [Appendix File A]. Of these 471 patients, 81 (17.2%) reported a poor outcome at the last follow-up contact. Multivariate Cox regression showed that patients with isolated myelopathy reported significantly poorer outcomes than those with isolated radiculopathy [Fig. 4]. A poor outcome in the long term was not significantly associated with the development of new symptoms, revision surgery at the index level, nor with additional adjacent segment surgery for CASP. It should be noted that the CASP patients did require additional surgery to reach a similar clinical outcome.

#### 3.3.4 Re-operation at index level

A total of 18 patients underwent re-operation at the index level; 15 were due to recurrent complaints, 2 were due to a post-operative hematoma and 1 was due to symptomatic cage subsidence. Of these 18 patients, re-ACD(F) was performed in 10 patients, dorsal foraminotomy in 6, while the remaining were treated with a corpectomy (n ​= ​1) and a laminectomy (n ​= ​1).

## 4 Discussion

There is increasing evidence that accelerated radiological degeneration takes place in levels adjacent to fused segments (Garrido et al., 2011; Robertson et al., 2005; Kim et al., 2009; Matsumoto et al., 2010). However, the role of fusion surgery at the previously operated segment(s) in the development of CASP remains controversial. This study provides insight into the incidence of CASP and additional adjacent segment surgery rates after anterior surgery for radiculopathy and/or myelopathy due to CDDD. We report a 9.7% incidence of CASP in a retrospective cohort of 601 patients with a mean follow-up of 5.0 years. We observe an incidence rate of 6.9% additional adjacent segment surgeries due to CASP (N ​= ​41). This corresponds to an annual rate of 1.4%, which is slightly lower than previously reported in the literature (Riew et al., 2012; Lawrence et al., 2012). Half of the additional surgeries were performed within 2.5 years, which suggests a peak incidence in the first years following the initial surgery.

Our findings show that pre-operative degeneration was similar between patients with and without CASP at the index level, as well as at the adjacent levels above or below. This argues against the course of natural degeneration being the only factor in the development of CASP, as suggested by previous literature (Hilibrand et al., 1999; Helgeson et al., 2013; Donnally et al., 2018; van Eck et al., 2014). In a multivariate analysis, baseline covariates did not significantly vary between patients with and without CASP. Therefore, baseline degeneration is unlikely to have a strong influence on the development of CASP.

The primary goal of ACD(F) is the relief of symptoms of radiculopathy and/or myelopathy through decompression of neural structures. Fusion in itself is not a requisite to reach this goal. This is reinforced by the fact that most studies report no correlation between bony fusion and clinical outcomes (Noordhoek et al., 2019). Dorsal foraminotomy does not induce fusion and is therefore suitable as a non-fusion control group, as was done in the FACET trial (Broekema et al., 2017). The additional adjacent segment surgery rate after dorsal foraminotomy is 2.9% in a 7-year follow-up, which is substantially lower than reported after fusion surgery (Clarke et al., 2007). Hence, there might be a role of fusion surgery in the accelerated development of CASP.

Due to the low number of available post-operative images and different imaging techniques used, a reliable assessment of radiological fusion could not be made in this study. Moreover, post-operative images have been made upon indication, as it is not the standard care in our center. This could result in selection bias, and possibly a type II error due to the smaller subset of patients. Finally, there is a large difference in time-span between index surgery and post-operative imaging (e.g. 12 months to >8 years), as indicated by the standard deviation of 3.6 years.

Anterior cervical discectomy with arthroplasty (ACDA) was developed in an effort to reduce the incidence of CASP, by preserving motion in the operated segment(s) (Korinth, 2008; Cummins et al., 1998). After a 7-year follow-up period, additional adjacent segment surgery rates for ACDA vary between 3.7% and 4.4% for single- and multilevel surgeries, respectively (Radcliff et al., 2017). For ACDF, they vary from 13.6% to 16.2% for single- and multilevel surgery, respectively (Radcliff et al., 2017). This is also confirmed by a recent study with a 10-year follow-up (Kim et al., 2021). These differences cannot be explained by natural degeneration alone, suggesting that fusion surgery plays an accelerating role in CASP development. ACD(F) results in high rates of fusion: 70–80% for ACD and 95–100% for ACDF with or without plating (Gore and Sepic, 1998; Dowd and Wirth, 1999; Fountas et al., 2007; Persson et al., 1997; Katz et al., 1997; Grob, 1998). In our population, a significant proportion of ACD patients developed CASP in comparison to ACDF patients. A possible explanation could be that the absence of an intervertebral implant may cause sagittal imbalance. An increased (segmental) kyphotic alignment after ACD is described in multiple studies (Xie and Hurlbert, 2007; Martins, 1976; Hauerberg et al., 2008; Van Den Bent et al., 1996; Pointillart et al., 1995; Haden et al., 2005; Savolainen et al., 1998).

Therefore, we studied the sagittal alignment from C2–C7 and the index level in our cohort. Due to lack of standardization of X-ray imaging for the measurement of sagittal alignment, comparability and reliability of angels seems to be questionable. Postural differences can explain the controversial findings presented in our study. Our impression is that standardization of imaging should be obligatory in analyzing cervical alignment, otherwise comparability is debatable. Despite the aforementioned shortcomings concerning the available imaging, data analysis was performed and showed no substantial differences between groups.

The ACDF patients that developed CASP specifically appeared to have significantly less C2–C7 lordosis after the index surgery, compared to those that did not develop CASP. Cervical sagittal balance as a whole, presented by C2–C7 lordosis, could thus be of influence on the development of CASP.

There was a statistically significant difference in segmental alignment of the index level between those with and without CASP, however the difference is small and not clinically relevant. We suspect that this difference is the consequence of a measuring error, especially since the segmental angles are smaller and thus more prone to errors.

The yearly incidence rate of additional adjacent segment surgeries after ACDF with stand-alone cage in our population is 0.8% (25/601 in 5.0 years), which is notably lower than the reported rate of 2.3–7% after ACDF with plate after 2–5 years (Radcliff et al., 2016; JE et al., 2013; PD et al., 2013). Previous research also shows lower rates of both RASP and CASP after ACDF with stand-alone cages in comparison to ACDF with plate-constructs (Xie and Hurlbert, 2007; Joo et al., 2010; Ahn et al., 2016; Ji et al., 2015). Interestingly, reported fusion rates are similar for ACDF with stand-alone cages (93.5%) and ACDF with plating (98%) (Mobbs et al., 2007). The small difference is not expected to have an influence on the incidence of CASP, as both techniques equally restore segmental alignment (Xie and Hurlbert, 2007). Differences in CASP between the two techniques might be explained by strain on the adjacent segments caused by the plate, or more extensive surgical preparation to accommodate the plate, hence increasing the chance of damaging the adjacent level. It has been proven that the disc height of adjacent levels significantly decreases in ACDF with plate constructs in comparison with ACDF with stand-alone cages (Joo et al., 2010). Another contributing factor might be the occurrence of subsidence of the plate-construct into the adjacent segment. Similarly, a common concern in ACDF with stand-alone cages is the high incidence of cage subsidence. However, reported subsidence rates vary; some studies show little to no difference in symptomatic subsidence between ACDF and ACDF with plates (Xie and Hurlbert, 2007; Joo et al., 2010). In our study, only 1 out of 601 patients (0.17%) underwent revision surgery due to symptomatic cage subsidence. We did not analyze fusion rates and radiologic subsidence in this retrospective study, since post-operative imaging is not standard in our center.

Clinical outcomes such as pain and satisfaction after anterior cervical surgery for CDDD are similar for all techniques at short-term follow-up, but are known to decline at long-term follow-up (Goffin et al., 2002; Nandoe Tewarie et al., 2007). For example, Nandoe Tewarie et al. described a decline when comparing short-term outcomes (2 months) with long-term outcomes (7 years). In the former, 90.1% satisfactory results were reported, while this dropped to 67.6% in the latter (Nandoe Tewarie et al., 2007). This is also described by Goffin et al. who reported clinical deterioration in 36% of patients at a 6-year follow-up in patients undergoing ACDF (Goffin et al., 2004). This is in line with our results, as we observed a deterioration of clinical outcomes when comparing the standard 6-week outpatient visit with the time of last follow-up [Appendix File A]. A poor result at the first post-operative outpatient visit (6 weeks) was significantly associated with the indication for surgery being myelopathy, and the pre-operative duration of complaints. This correlated with a duration of symptoms longer than 3 months for those with radiculopathy, and longer than 6 weeks in myelopathy. For patients with radiculopathy, satisfactory results declined from 93% to 90.5%, and for patients with myelopathy, the reported decline went from 88.4% to 70.2%, after a mean of 5.0 years.

The main limitation of this study is its retrospective design. Specifically, not all data could be retrieved from the patient charts, and there is no comparative group. The main missing data were KSs, as not all pre-operative X-rays were available, or C7 was overshadowed by the shoulders. A missing value analysis showed complete random distribution of these missing values, which decreases the chance of bias. The preferred surgical technique in our center is ACDF with stand-alone cages, which may limit the generalizability of our findings, as it is not standard in other parts of the world. Moreover, the relatively small sample size of patients with ACDF with plate constructs (N ​= ​48) and ACD (N ​= ​23) might skew our results. Nevertheless, we decided to include these in the study, as the intent was to analyze all anterior decompression surgeries, as predefined in our protocol. However, we decided to remove the corpectomy patients (N ​= ​2) for this reason. Although the average follow-up time of 5.0 years might not be long enough to identify all CASP cases, our data indicated that the majority of CASP manifests in the first 2.5 years, hence the average follow-up time of 5.0 is indeed sufficient to provide important insight into this controversial issue. Another limitation is the assessment of fusion in this study*.* Post-operative imaging was available in only 127 of 601 cases, which entail variable imaging techniques. In some cases, fusion status was scored “inconclusive” due to the suboptimal imaging techniques used. The same limitations apply for the assessment of cervical sagittal balance: very few images were available, and were taken at different time-points in relation to the index surgery and often made upon indication, without any standardization.

Moreover, measurements to assess the clinical outcomes of patients are subjective and cannot be confirmed objectively in this retrospective study design. Of the 130 patients (21.6%) that were not reached by telephone, a re-operation due to CASP in another hospital may have been missed. However, we judge this risk to be small, as only one patient of the 471 contacted patients underwent an additional adjacent segment surgery in another hospital.

## 5 Conclusion

In this retrospective cohort, we observe a relatively low rate of additional surgery for CASP in ACDF with stand-alone cages. The majority of additional surgeries for CASP took place in the first 2.5 years following index surgery. Moreover, patients with CASP did not have a higher degree of baseline degeneration. We suggest that surgical technique, fusion, segmental kyphosis and natural degeneration play a multifactorial role in the development CASP. Complication rates were low and clinical outcomes were similar for all techniques used. This study gives insight in the incidence and accelerating factors of CASP in our daily practice.

## Funding

The authors did not receive support from any organization for the submitted work.

## Ethics approval

This study has been approved by the Medical Ethical Committee (METCZ20200004) and was conducted according to principles enshrined in the Declaration of Helsinki and in accordance with the Medical Research Involving Human Subjects Act (WMO).

## Data availability

The datasets generated during and/or analyzed during the current study are available from the corresponding author on reasonable request.

## Declaration of competing interest

The authors declare that they have no known competing financial interests or personal relationships that could have appeared to influence the work reported in this paper.
