# Supplementary material for: Clinical adjacent segment pathology after anterior cervical discectomy, with and without fusion, for cervical degenerative disc disease: A single center retrospective cohort study with long-term follow-up
Source: Brain Spine. 2022 Jan 22;2:100869. doi: 10.1016/j.bas.2022.100869 (PMC9560678; doi:10.1016/j.bas.2022.100869)
Supplement: Multimedia component 1 [file mmc1.docx]

|  | | **Total**  **(N=601)** | **No CASP**  **(N=560)** | | **CASP**  **(N=41)** | **p-value** |
| --- | --- | --- | --- | --- | --- | --- |
| **Radiculopathy** | | *N=344* | *N=323* | | *N=21* |  |
|  | Good | 320 (93.0%) | 301 (93.2%) | | 19 (90.5%) | 1.000 |
|  | Poor | 21 (6.1%) | 20 (6.2%) | | 1 (4.8%) |  |
|  | Unknown | 3 (0.9%) | 2 (0.6%) | | 1 (4.8%) |  |
| **Myelopathy** | | N=207 | *N= 192* | | *N=15* |  |
|  | Good | 183 (88.4%) | 170 (88.5%) | | 13 (86.7%) | 1.000 |
|  | Poor | 19 (9.2%) | 18 (9.4%) | | 1 (6.7%) |  |
|  | Unknown | 5 (2.4%) | 4 (2.1%) | | 1 (6.7%) |  |
| **Myeloradiculopathy** | | *N=50* | *N=45* | | *N=5* |  |
| *Radiculopathy complaints* | Good | 45 (90%) | 40 (88.9%) | | 5 (100%) | 1.000 |
|  | Poor | 5 (10%) | 5 (11.1%) | | 0 (0%) |  |
| *Myelopathy complaints* | Good | 47 (94%) | 43 (95.6%) | | 4 (80%) | 0.196 |
|  | Poor | 2 (4%) | 1 (2.2%) | | 1 (20%) |  |
|  | Unknown | 1 (2%) | | 1 (2.2%) | 0 (0%) |  |

**Appendix A, Table 1: Short-term clinical outcomes.**

This table represents the PROMs indicated by patients at their last outpatient visit concerning the initial surgery. For radiculopathy, a clinical outcome was only considered good if improvement of the pre-operative complaints occurred. As for myelopathy, a clinic outcome was also considered good if stabilisation of complaints was achieved. Some patients reported mixed complaints of myeloradiculopathy which could not be differentiated from chart review, these are represented separately. Subgroups of those having with- and without additional surgery for CASP are represented, significance is calculated using Fisher’s Exact test with a Bonferroni correction.

| **Total group** | **Odom's Criteria** | **Total**  **(N=471)** | **No CASP**  **(N=438)** | **CASP**  **(N=33)** | **p-value** |
| --- | --- | --- | --- | --- | --- |
|  | Excellent | 166 (35.2%) | 158 (36.1%) | 8 (24.2%) | 0.566 |
|  | Good | 132 (28.1%) | 122 (27.9%) | 10 (30.3%) |  |
|  | Satisfactory | 92 (19.5%) | 84 (19.2%) | 8 (24.2%) |  |
|  | Poor | 81 (17.2%) | 74 (16.9%) | 7 (21.2%) |  |
| **Subgroups** | | |  |  |  |
| **Radiculopathy** | | **N=274** | **N=256** | **N=18** |  |
|  | Excellent | 126 (46.0%) | 119 (46.5%) | 7 (38.9%) | 0.821 |
|  | Good | 74 (27.0%) | 69 (27.0%) | 5 (27.8%) |  |
|  | Satisfactory | 48 (17.5%) | 44 (17.2%) | 4 (22.2%) |  |
|  | Poor | 26 (9.5%) | 24 (9.4%) | 2 (11.1%) |  |
| **Myelopathy** | | **N=151** | **N=140** | **N=11** |  |
|  | Excellent | 27 (17.9%) | 26 (18.6%) | 1 (9.1%) | 0.922 |
|  | Good | 45 (29.8%) | 41 (29.3%) | 4 (36.4%) |  |
|  | Satisfactory | 34 (22.5%) | 31 (22.1%) | 3 (27.3%) |  |
|  | Poor | 45 (29.8%) | 42 (30.0%) | 3 (27.3%) |  |
| **Myeloradiculopathy** | | **N=46** | **N=42** | **N=4** |  |
|  | Excellent | 13 (28.2%) | 13 (31.0%) | 0 (0%) | 0.422 |
|  | Good | 13 (28.3%) | 12 (28.6%) | 1 (25%) |  |
|  | Satisfactory | 10 (21.7%) | 9 (21.4%) | 1 (25%) |  |
|  | Poor | 10 (21.7%) | 8 (19.0%) | 2 (50%) |  |

**Appendix A, Table 2: Long-term clinical outcomes.**

This table represents the Odom’s Criteria of patients that were reached for long-term follow-up. The total group is represented, as well as subgroups for the indication of surgery and those with- and without CASP as this proved significantly different in multivariate analysis. Significance between subgroups was determined with Chi-Square and/or Fisher’s exact test with Bonferroni correction.
